# Defining Compulsive Behavior

**DOI:** 10.1007/s11065-019-09404-9

**Published:** 2019-04-23

**Authors:** Judy Luigjes, Valentina Lorenzetti, Sanneke de Haan, George J. Youssef, Carsten Murawski, Zsuzsika Sjoerds, Wim van den Brink, Damiaan Denys, Leonardo F. Fontenelle, Murat Yücel

**Affiliations:** 1 Department of Psychiatry, Amsterdam UMC, University of Amsterdam, PA3.227, PO Box 22660, 1100DD Amsterdam, The Netherlands; 2 Brain Imaging Center, Academic Medical Center, Amsterdam UMC, Amsterdam, The Netherlands; 3 Psychological Sciences, University of Liverpool, Liverpool, UK; 4 Culture Studies, Tilburg University, Tilburg, The Netherlands; 5 School of Psychology, Deakin University, Geelong, Australia; 6 Murdoch Childrens Research Institute, Population Studies of Adolescents, The Royal Children’s Hospital Melbourne, Parkville, Australia; 7 Department of Finance, The University of Melbourne, Parkville, Victoria Australia; 8 Institute of Psychology, Cognitive Psychology Unit & Leiden Institute for Brain and Cognition, Leiden University, Leiden, The Netherlands; 9 Department of Neurology, Max-Planck Institute for Human Cognitive and Brain Sciences, Leipzig, Germany; 10 Netherlands Institute for Neuroscience, An Institute of the Royal Netherlands Academy of Arts and Sciences, Amsterdam, The Netherlands; 11 Obsessive, Compulsive, and Anxiety Spectrum Research Program, Institute of Psychiatry, Federal University of Rio de Janeiro, Rio de Janeiro, Brazil; 12 D’Or Institute for Research and Education, Rio de Janeiro, Brazil; 13 Turner Institute for Brain and Mental Health, School of Psychological Sciences, Monash University, Melbourne, Australia

**Keywords:** Compulsivity, Definition, Phenomenology, Observational perspective

## Abstract

Compulsive tendencies are a central feature of problematic human behavior and thereby are of great interest to the scientific and clinical community. However, no consensus exists about the precise meaning of ‘compulsivity,’ creating confusion in the field and hampering comparison across psychiatric disorders. A vague conceptualization makes compulsivity a moving target encompassing a fluctuating variety of behaviors, which is unlikely to improve the new dimension-based psychiatric or psychopathology approach. This article aims to help progress the definition of what constitutes compulsive behavior, cross-diagnostically, by analyzing different definitions in the psychiatric literature. We searched PubMed for articles in human psychiatric research with ‘compulsive behavior’ or ‘compulsivity’ in the title that focused on the broader concept of compulsivity—returning 28 articles with nine original definitions. Within the definitions, we separated three types of descriptive elements: phenomenological, observational and explanatory. The elements most applicable, cross-diagnostically, resulted in this definition: Compulsive behavior consists of repetitive acts that are characterized by the feeling that one ‘has to’ perform them while one is aware that these acts are not in line with one’s overall goal. Having a more unified definition for compulsive behavior will make its meaning precise and explicit, and therefore more transferable and testable across clinical and non-clinical populations.

## Introduction

In many psychiatric or psychopathological disorders, patients repetitively engage in behaviors that are disruptive for themselves and their environment. In obsessive-compulsive disorder (OCD), patients often report that they realize the nonsensical nature of their compulsions (e.g., washing hands 20 times) and the disruptive effects the compulsions have on their life, but cannot always give a reason for why they do them (Denys, 2011). Addicted patients can often explain why they started using the substance of abuse or betting on electronic gaming machines (e.g., pleasure, peer pressure, stress relief). At a certain stage of the addiction process, however, these reasons no longer drive the behavior and many patients struggle to understand why they keep using despite an increasing awareness of the destructive impact of the substance or behavioral addiction (Kennett, Matthews, & Snoek, 2013).

Although the behaviors themselves differ in many aspects across these disorders, there seems to be a motivational drive behind them all that defies simple explanations. Patients feel compelled to act out these behaviors—from now on referred to as ‘compulsive behavior.’ A range of compulsive behaviors cut across different diagnostic boundaries, including substance and behavioral addictions, obsessive-compulsive and related disorders, eating disorders, and neurological disorders such as Parkinson’s disease (Allen, King, & Hollander, 2003; Flessner, Knopik, & McGeary, 2012; Grant & Potenza, 2006; Le Moal & Koob, 2007; Leeman & Potenza, 2012; Rothemund et al., 2011). Compulsive behaviors are relevant even beyond psychiatric disorders. In the general population, about 10% of people have OCD-related sub-threshold symptoms that include compulsive behaviors, and some authors have even suggested that normal repetitive daily behaviors such as children’s bedtime rituals have compulsive elements (Denys, 2014; Fineberg et al., 2013).

A question then arises—does the shared phenomenology of compulsive behaviors across disorders indicate that there are shared cognitive and neural mechanisms driving them? If there are, investigating these mechanisms would be important for many disorders in psychiatry and psychology and may lead to new avenues for cross-diagnostic treatments. On the other hand, the types of behavior that become compulsive differ greatly between disorders, and investigating them as one cross-diagnostic construct could lead to generalizations that may hamper rather than advance our understanding. Precisely defining compulsive behavior is a fundamental first step in solving this issue. To investigate compulsive behavior as a cross-diagnostic construct, we need a clear notion of which behaviors will be included and excluded. In this paper, we will first explain and explore this issue in the introduction, and then conceptualize a clear definition of compulsive behavior by searching the literature and comparing and discussing different definitions used in the field of behavioral sciences.

### Compulsivity as a Cross-Diagnostic Behavioral Dimension

The idea that compulsivity is an important behavioral dimension that needs to be investigated across disorders is gaining popularity, and over the last few years, studies into compulsivity have increased in number (Robbins, Gillan, Smith, de Wit, & Ersche, 2012). A PubMed search shows that the number of papers with ‘compulsivity’ in the title or summary more than doubled in the last five years (*n* = 236 in 2011–2015) compared with the previous five years (*n* = 97 in 2006–2010) and multiplied by four compared with the five years prior to that (*n* = 52 in 2001–2005). Moreover, the Strategic Plan of the U.S. Institute of Mental Health (NIMH) aims to establish a new classification of psychiatry for research purposes based on dimensions of observable behavior and neurobiological measures instead of diagnostic categories (National Institute of Mental Health Strategic Plan, 2008). The current interest in such a new classification results from the discontent with the progress of neurobiological research in psychiatry and the idea that these behavioral dimensions may map more closely to findings from neurobiological research. Compulsive behavior is included as one of these behavioral dimensions in the NIMH’s Research Domain Criteria (RDoC), underlining the interest in research community in investigating compulsive behavior as a cross-diagnostic dimension in psychopathology (Cuthbert, 2014).

Several studies suggest that shared cognitive and neural mechanisms underlie compulsive behaviors across disorders. A compulsive brain circuitry has been suggested based on overlapping neural circuits that underlie compulsive behaviors in different psychiatric disorders (Fontenelle, Oostermeijer, Harrison, Pantelis, & Yücel, 2011; van den Heuvel et al., 2016). These circuits are associated with cognitive processes implicated in the development of compulsive behaviors. Among the most replicated findings in compulsive disorders are abnormalities in the frontostriatal circuit (Baxter et al., 1992; Robinson et al., 1995; van den Heuvel et al., 2010). Regions in this circuit, such as the orbitofrontal cortex and ventral striatum, are involved in reinforcement learning, steering behavior and—especially in the more dorsal region of the striatum—repetitive performance of learned behavior (Shohamy, 2011). Disturbances in learning processes (e.g. increased errors or reaction times in a reversal learning task or differences in sensitivity to learning from positive versus negative outcomes) and associated fronto-striatal circuits may contribute to the inflexibility and persistence that characterize compulsive behaviors (de Ruiter et al., 2009; Remijnse et al., 2006; Valerius, Lumpp, Kuelz, Freyer, & Voderholzer, 2008). Functional deviations in regions of cognitive control such as the inferior frontal gyrus, dorsolateral prefrontal cortex (PFC), anterior cingulate cortex (ACC), and presupplementary motor area have been associated with the inability to overcome and regain control over compulsive behaviors despite destructive consequences. Moreover, preventing negative outcomes such as anxiety, uncertainty, or stress-like states may be an important motivating factor behind compulsive behaviors. A popular dual processing framework that encapsulates these neural cognitive abnormalities, suggests compulsive behavior involves an imbalance between hypoactive goal-directed control and hyperactive habit learning systems, with obsessions and trait anxiety playing a role as an independent contributor to compulsive avoidance habit learning (Fineberg et al., 2018; Gillan, Robbins, Sahakian, van den Heuvel, & van Wingen, 2016). Where either an overreliance on habitual learning (i.e., strong stimulus response links) associated with abnormalities in the putamen or reduced goal-directed control associated with abnormalities in the caudate and ventromedial PFC result in the inflexibility and persistence that characterizes compulsive behaviors. However, also within this framework it is possible that different types of compulsive behavior are coupled to specific disturbances of the goal-directed control and habit learning systems (e.g., one type of compulsivity might be more related to goal-directed control deficits whereas another type to excessive habitual learning).

While the dysfunctional cognitive constructs and neural regions might be similar across many disorders, the underlying nature of these neural and cognitive processes may be diametrically different. For instance, although abnormalities in similar regions have been found in OCD and addiction, the direction of the deviation with healthy controls can be entirely opposed. In OCD patients, error- or conflict-related ACC hyperactivation has been found, while in addictions, ACC hypoactivation was found during error processing (Luijten et al., 2014; Melcher, Falkai, & Gruber, 2008). Although both disorders may have a suboptimal response to errors—which could lead to aberrant learning and decision-making underlying compulsive behaviors—the mechanisms behind this affected function may differ. Moreover, the point at which compulsive behaviors develop in the disease trajectory can differ between disorders, which may point at different mechanisms underlying compulsive behavior development. In addiction, behavior typically becomes more compulsive in later stages of the disorder, while compulsive behaviors characterize OCD from early on in the disorder (Coles, Johnson, & Schubert, 2011; Koob, 2009). In anorexia nervosa, it is difficult to establish whether behaviors related to food-restriction or excessive exercise start out as compulsive or develop later. At onset, these behaviors are driven both by the reinforcing effect of accomplishment (weight loss) as well as by preventing negative outcomes (e.g., negative feelings associated with weight gain). The initial behavior could be viewed as reward-driven similar to the first stages of addiction or could also be interpreted as a rigid type of avoidance behavior similar to OCD symptoms (Godier & Park, 2014). While in trichotillomania it may differ greatly between patients, in some cases hair pulling may start out as a pleasurable activity, while for others it may feel compulsive right from the start. Therefore, while there are striking similarities between these compulsive behaviors (e.g., repetitive and destructive, overlap in neural circuits), the nature of the compulsive behaviors across different disorders can differ significantly regarding the role of positive or negative reinforcement, level of goal-directedness, developmental trajectory, and direction of neural abnormalities.

### Lack of Definition for Compulsivity

This brings us back to our important question of whether the overlap of compulsive behaviors between disorders is substantial enough to justify investigating compulsivity as a cross-diagnostic construct. Difference between disorders in the developmental trajectories and mechanisms that lead to compulsive behaviours may be too large to be usefully isolated and investigated. Perhaps it is more useful to divide and study compulsive behavior subtypes separately. The main hurdle for investigating this question is that we have no agreed-upon definition of the concept of compulsivity, as reflected in the wide variety of definitions in Table 1 (Denys, 2014; Yücel & Fontenelle, 2012). This lack of consensus creates confusion and raises the possibility that the term ‘compulsivity’ refers to different constructs within and across psychiatric disorders. This confusion is also reflected in the lack of agreed-upon measurement of compulsivity such as a questionnaire or a neuropsychological test beyond those intertwined with certain psychiatric diagnoses (e.g., Yale–Brown Obsessive Compulsive Scale; Goodman et al., 1989).

**Table 1:** Note: This data is mandatory. Please provide

Author (year)	Descriptions compulsivity/ compulsive behaviour	Phenomenological	Observational	Explanatory
Torregrossa, Quinn, and Taylor (2008)	With compulsivity a person feels compelled to perform a behaviour in order to relieve anxiety or stress, even if the behaviour is inappropriate or counterproductive	(C) a person feels compelled to perform a behaviour	(B) even if the behaviour is inappropriate (C) or counterproductive	(C) in order to relieve anxiety or stress
Fineberg et al. (2010)	Compulsivity refers to a tendency to perform unpleasant repetitive actions in a habitual or stereotypical fashion in order to prevent perceived negative consequences leading to functional impairment	(D) unpleasantly repetitive actions	(A) the tendency to perform repetitive acts (D) in a habitual or stereotyped manner (C) leading to functional impairment	(A) prevent perceived negative consequences
Dalley et al. (2011)	Compulsivity are actions inappropriate to the situation which persist, have no obvious relationship to the overall goal and which often result in undesirable consequences		(B) actions inappropriate to the situation (A) which persist (B) have no obvious relationship to overall goal (C) Which often result in undesirable consequences	
Denys (2014)	Inability to not perform an act, with a subjective feeling of loss of control vis-à-vis oneself	(A) inability to not perform an act (B) subjective feeling of loss of control vis-à-vis oneself		
Figee, Wielaard, Mazaheri, and Denys (2013)	Compulsivity encompasses the repetitive, irresistible urge to perform a behavior, the experience of loss of voluntary control over this intense urge, the diminished ability to delay or inhibit thoughts or behaviors and the tendency to perform repetitive acts in a habitual or stereotyped manner	(C) irresistible urge to perform a behaviour (B) the experience of loss of voluntary control over this intense urge (A) the diminished ability to delay or inhibit thoughts or behaviors	(A) the tendency to perform repetitive acts (D) in a habitual or stereotyped manner	
Berlin and Hollander (2014)	Compulsivity can be characterized by perseverative, repetitive actions that are excessive and inappropriate to a situation		(A) perseverative, repetitive actions (B) excessive and inappropriate to a situation	
Fineberg et al. (2014)	Compulsivity represents the performance of repetitive and functionally impairing overt or covert behavior without adaptive function, performed in a habitual or stereotyped fashion, either according to rigid rules or as a means of avoiding perceived negative consequences		(A) performance of repetitive (C) and functionally impairing behaviour (B) without adaptive function (D) performed in a habitual or stereotyped fashion	(B) either according to rigid rules (A) or as a means of avoiding perceived negative consequences
Gillan, Robbins, Sahakian, van den Heuvel, and van Wingen (2016)	Compulsivity, which we define broadly as a loss of control over goal-directed behavior	(B) loss of control over goal-directed behavior		
Grant et al. (2016)	Compulsivity is characterized by actions which are persistently repeated despite adverse consequences		(A) actions which are persistently repeated (C) despite adverse consequences	

Moreover, the frontostriatal circuitry is implicated in many cognitive processes including decision-making, motivation, salience and cognitive control, and is also disturbed in psychiatric disorders not typically associated with compulsive behavior, such as schizophrenia. Therefore, the observation that all compulsive disorders share abnormalities in the frontostriatal circuitry is not necessarily indicative of a shared neural mechanism specific to compulsivity. Without a definition, we cannot expect much progress from compulsivity research within psychiatry’s new dimension-based approach. A vague conceptualization will make compulsivity a moving target encompassing a fluctuating variety of behaviors, which is unlikely to improve the biological footing of psychiatry or psychopathology research.

The word ‘compulsive’ has been used for years both informally and in clinical practice. Its use shapes its meaning. In this review, we aim to comprehensively document how this term has been used in the behavioral sciences field and synthesize this into a common understanding of compulsivity that we can use to classify behavior in a coherent way and distinguish it from other types of pathological behaviors. We aim to conceptualize compulsive behavior in a broad manner that is inclusive of its use in different disorders.

## Methods

### Literature Search

We searched PubMed for relevant articles by using the key words ‘compulsive behavior’ or ‘compulsivity.’ Papers having the above key words on their title were searched for definitions of compulsivity or compulsive behavior. In order to avoid definitions of compulsivity found in articles that were merely intended for a specific disorder or situation, we excluded papers that investigated one or two specific disorders. Therefore, the resulting articles are all focused on compulsivity in a broader sense. It is useful to note that these articles were not intended to provide a comprehensive definition of compulsivity, although they give a good idea of how compulsivity is used in the literature. We also excluded articles focusing only on animal research.

This search and selection strategy resulted in 28 articles: 15 included a definition of compulsivity or compulsive behavior, 10 gave no definition of compulsivity or compulsive behavior and three discussed or described compulsivity without specifically defining it (Robbins, Curran, & de Wit, 2012; van den Heuvel et al., 2010; Yücel & Fontenelle, 2012). Of the 15 papers that included a definition of compulsivity or compulsive behavior, six used a previous definition reported in another article from the list, leaving nine original definitions (see Table 1). Although some of the definitions overlap and are sometimes used by the same research groups, we will discuss them separately.

### Elements of Compulsivity in Definitions

We listed the original definitions and divided them into three description types: phenomenological, observational and explanatory. Respectively, these describe the actor’s experience during the behavior, how the behavior can be identified from a third-person perspective, and what motivation drives the behavior. We used these description types to dissect the concepts found in the literature.

## Results

### Phenomenological Elements

Five papers include phenomenological descriptions in their definition, leading to four phenomenological elements: (A) *inability not to perform an act*, (B) *experienced loss of control*, (C) *feeling one ‘has to’ perform an act*, (D) *unpleasantness of repetitive actions* (see Table 1). Elements A, B and C are well described in an article by Denys (2014). The author looked at different behaviors associated with compulsivity to find the essence of compulsivity and stripped them of elements not present in all forms of compulsivity. What is left—common to all manifestations across psychiatric disorders—is the experience that one *has to* or *feels (internally) forced to* perform an act. Or in other words, one feels *unable not to perform* the act (Denys, 2014). This leads to the experience of loss of control. Figee et al. (2013) describe the feeling of *having to act* physically as an *irresistible urge to act* with loss of control over this urge, and additionally as the *experienced inability (or diminished ability) not to* act. This is in accordance with the phenomenological element of Torregrossa et al. (2008) that a person feels compelled to perform a behavior. Another paper uses only element B to define compulsive behavior, namely, a loss of control over goal-directed behavior (Gillan et al., 2016). And the last paper describes compulsive actions as unpleasant (D: Fineberg et al., 2010).

### Observational Elements

Seven papers include descriptive aspects of compulsive behavior, resulting in four observational elements: (A) behavior is *repetitive*, (B) it is *nonadaptive or inappropriate for the context*, (C) it *leads to functional impairment*, (D) it is *performed in a habitual or stereotyped manner*. Four of these papers describe compulsive behavior as ongoing (i.e., repetitive or persisting) despite negative consequences (e.g., leading to functional impairment: Dalley, Everitt, & Robbins, 2011; Fineberg et al., 2014, 2010; Grant et al., 2016), while three alternatively or additionally state that compulsive behaviors lack a clear function (i.e., inappropriate for context or without adaptive function or no obvious relationship to overall goal, aspect B: Berlin & Hollander, 2014; Dalley et al., 2011; Fineberg et al., 2014). One paper (Torregrossa et al., 2008) does not specify that the behavior is ongoing but does include that the behavior is performed even if inappropriate (B) or counterproductive (C). Two of the papers describe that compulsive behavior is performed in a habitual or stereotyped fashion (D: Figee et al., 2013; Fineberg et al., 2014).

### Explanatory Elements

Three papers provide three different elements that give explanatory descriptions of compulsive action: (A) *to prevent negative consequences*, (B) *according to rigid rules*, (C) *to reduce anxiety or stress*. Two definitions state that the behavior is performed to prevent perceived negative consequences, one of which provides an alternative possibility, namely, to prevent perceived negative consequences or according to rigid rules (Fineberg et al., 2014, 2010). Finally, Torregrossa et al. (2008) state that the behavior is performed to relieve anxiety or stress.

## Discussion

In accordance with Denys (2014), we consider the feeling that one *has to* perform an action, or the inability to stop performing an action, as the most essential characteristic of compulsive behavior present in all forms of compulsivity. This core aspect is necessary to label a behavior compulsive and is associated with lack of control over that specific behavior. The feeling that one has to perform an action or is not capable of stopping an action indicates that that behavior, at least partially, diverges from volitional control. For instance, we say that addiction turns compulsive with the experience that the behavior is less steered by one’s will and more by an uncontrollable internal force (Koob, 2017; Solomon, 1980; Solomon & Corbit, 1974). The label compulsive therefore implies an internal struggle, that is, one feels as if one *has to* perform a behavior irrespective of one’s will. Three phenomenological elements point at this: (A) the inability not to perform an act, (B) experienced loss of control, (C) feeling one *has to* perform an act.

Augmenting the phenomenological description, the observational elements describe how the behavior appears from an outside perspective. The description that one feels one *has to* perform the behavior may be necessary to establish whether the behavior is compulsive but may lack in specificity and thus not be sufficient. For instance, a behavior only performed once may be felt as if one *has to* perform it, yet we would not call a once-off act compulsive. Additional observational elements may clarify what the behaviors we usually label as compulsive have in common besides the phenomenological experience.

Two observational elements, (1) repetitive behavior and (2) resulting in negative consequences, were used by most of the papers and respective definitions in Table 1 (in six and five of nine definitions, respectively), often in combination (four of nine definitions). Element 2 is augmented or alternated in four definitions with the element that behavior is inappropriate for its context. That the behavior is inappropriate or results in negative consequences emphasizes the dysfunctional nature of compulsive behavior and implies that the behavior, from an outsider’s perspective, is not in line with the actor’s overall goals or at least conflicts with certain goals. This also describes the divergence between behavior and volition, but from an outsider’s perspective, namely, acting in a way that leads to negative consequences or results in no obvious valuable outcome. Additionally, the repetitive nature is the most frequently used element in the definitions, indicating its importance. Much of the compulsivity is in the persisting nature of the behavior.

Finally, the last observational element included in three of eight definitions is that the behavior is performed in a habitual or stereotyped fashion. This element may be included to emphasize that the behavior is repeated in a specific (or fixed) way. Compulsive behaviors in OCD, skin picking, or trichotillomania often have a fixed pattern. However, compulsive behaviors in eating disorders, hoarding disorder and addictions often vary and may not always be performed in a specific pattern. Therefore, this element seems less useful in a transdiagnostic conceptualization of compulsive behavior.

Three definitions (Berlin & Hollander, 2014; Dalley et al., 2011; Grant et al., 2016) include exclusively observational elements, which could be an advantage for translational research because no information is needed on phenomenological or motivational (explanatory) aspects. However, if the definition exclusively consists of observational elements, it implies that behavior could be labeled compulsive without the person experiencing it as such. For instance, if a person loses their job due to drug use, would we qualify it as compulsive even if they feel they *could* stop whenever they want but willingly took the risk of losing their job? If a person spends two hours a day turning a light switch on and off, would this be qualified as compulsive even if they enjoy the behavior and feel they *could* stop any time? In these examples the behaviors are repetitive and result in negative consequences or are inappropriate for the context. Therefore, based on exclusively observational criteria the two examples above would qualify as compulsive. However, we would argue that the label compulsive should be restricted to those instances where one feels forced or driven to act against one’s volition. If behavior and volition completely concur, one would *want to* perform the act and not experience *having to* perform the act. Moreover, the phenomenological element also distinguishes compulsive behavior from stereotyped behavior and habits, both of which can be repetitive and inappropriate, and lead to negative consequences. Therefore, observational elements alone do not suffice to capture the essence of human compulsive behavior.

Three definitions provide explanatory elements (describing the motivation behind the act). Two explanatory elements are: (1) to prevent or avoid perceived negative consequences or suffering (2) to reduce anxiety or stress. Negative consequences can be interpreted very widely and could include a specific event that is prevented (i.e., OCD patient washes hands to prevent contamination with disease), a feeling or state that is prevented (i.e., withdrawal symptoms or difficult emotions in addiction) or a vague ominous idea that ‘something bad’ may happen if the behavior is not performed. Element 2 is more specific and states that the behavior is aimed at reducing stress or anxiety, suggesting that it is a means of avoiding these already-present feelings or state. Whether decreasing anxiety or stress always directly motivates compulsive behavior is unknown. Even in OCD where anxiety commonly occurs, reported anxiety is variable between patients—and when it is present, it is hard to establish whether compulsions are driven by anxiety or may actually result in anxiety. This issue can be avoided by stating that behavior is *aimed at reducing or preventing a negative outcome* which includes both of the explanatory elements. Here a negative outcome can also include states or feelings such as stress or discomfort. This rephrasing of the first two explanatory elements is in line with the DSM diagnosis of OCD where these elements likely derive from. In OCD compulsions are aimed at preventing or reducing distress, or preventing some dreaded event or situation. Also, in anorexia nervosa compulsions are aimed at decreasing weight or preventing weight gain. Although it has been suggested that, over time, anorexia nervosa behaviors become inherently rewarding without weight loss, the idea of gaining weight still causes considerable distress to patients in the later stages of the disease (Godier & Park, 2014). Moreover, as previously mentioned, addiction is thought to enter a compulsive phase when it is driven more by alleviating a negative emotional state than pursuing a pleasurable state (Koob, 2009).

We think it is an interesting hypothesis that compulsive behavior is aimed at reducing or preventing a negative outcome and believe it may play an important role in much of the compulsive behavior across disorders. However, it is debatable whether every form of compulsive behavior is aimed at reducing or preventing a negative outcome. The above mentioned dual processing theory, views compulsive behavior as the result of excessive habit-formation and therefore mostly driven by reinforced stimulus–response contingencies (Gillan et al., 2016). Over time, patients may experience their behavior as more automatic and may not always be aware that anxiety or stress or the prospect of a negative event is driving the behavior. In the same line of reasoning compulsive behavior might not always be performed according to rigid rules, which is the third explanatory element. Therefore, we do not recommend including these explanatory elements in the definition at this time. However, we encourage future studies to investigate the extent to which compulsive behavior is generally driven by the aim to reduce or prevent a negative outcome.

On a side note, we would like to mention that the prevention of a negative outcome and habitual tendencies are not necessarily mutually exclusive explanations of compulsive behaviors. The combination of these two factors could very well give compulsive behaviors much of their inflexibility and persistence. The prevention or reduction of a negative outcome can become habitual over time––a habit of avoidance. While the person may be largely successful at keeping stress and anxiety at bay using compulsive acts, there is still an affective undertone driving these acts (Sjoerds, Luigjes, van den Brink, Denys, & Yücel, 2014). This can become apparent if the person is stopped from performing the act and stress levels rise. This seems to be how an OCD compulsion develops, often beginning as an aim to reduce or prevent a negative outcome but becoming more habitual and seemingly less affect-driven over time. The affective undertone is often exposed in response prevention therapy where OCD patients must resist the urge to perform the compulsive act. Many patients experience increased anxiety even if they were not aware of anxiety driving the compulsive behavior. In addiction, behavior is labeled compulsive when it shifts from being driven by rewarding properties to being driven by negative prospects (e.g., feelings of shame or withdrawal symptoms) or alleviating negative feelings (Koob, 2009). Also in addiction, the prevention or reduction of a negative outcome may turn habitual with repetition, however, at a later stage of the disorder.

### Definition of Compulsive Behavior

The phenomenological description that one feels as if one *has to* perform the behavior and the observational description that the behavior is repetitive and not in line with one’s overall goal both indicate that the behavior diverges from one’s will. When behavior diverges from one’s will, a feeling of control over the behavior is lost. However, to simply state that compulsive behavior is unwanted is problematic because at the moment of performing an action, one may feel as if one wants to perform the behavior (e.g., to reduce anxiety, relieve the urge) while also being aware that on another level (e.g., the results of the behavior) it is not what one wants. This is different from impulsive behavior where behavior and will can fully concur at the moment of action although one may later regret this action (e.g., buying a sports car that one cannot afford). Thus, in compulsive behavior, there is awareness at the time of performing an action that the behavior to some extent is unwanted. This goes together with the ongoing and dynamic internal struggle of compulsive behavior.

We suggest the following definition: compulsive behavior consists of repetitive acts that are characterized by the feeling that one *has to* perform them while one is aware that these acts are not in line with one’s overall goal. As mentioned at the beginning of the discussion, we view the feeling that one *has to* perform the act as the essence of compulsive behavior. In addition, to specify compulsive behavior further and distinguish it from behaviors with this same experience, we find it important to include that the behavior is persisting (repetitive) and is dysfunctional or unwanted. This is in line with the definitions in Table 1 that include inappropriateness or resulting in negative consequences (observational elements B and C in Table 1). However, since direct negative consequences are not always clear with all types of compulsive behavior (e.g., OCD compulsions may not result in harmful consequences outside time spent on them), and what is appropriate can be interpreted differently among people or cultures, we chose to formulate this dysfunctional nature as ‘not in line with one’s overall goal.’ This emphasizes that the behavior is unwanted in a broader perspective (i.e., it conflicts with one’s overall concerns, such as building a career or spending time with family) while keeping the possibility open that at the moment of the compulsive act, one may feel as if one wants to perform it (e.g., to relieve stress). We suggest adding to the definition of compulsivity that one is aware that these acts are not in line with one’s overall goals. This will help distinguish it from behavior that is performed in an impulsive or habitual manner, or behavior that others find dysfunctional while the person does not experience a conflict with their goals. The experience that someone feels internally forced to act out the behavior implies a level of resistance, knowing that to some extent one does not want to perform the act.

Therefore, behavior that aligns with the person’s goals, causing no resistance, conflict or experience of being forced to act, will not be called compulsive no matter how dysfunctional. We realize that in clinical practice, the experience of one’s behavior is rarely consistent. Sometimes patients experience the behavior in accordance with their goals while at other times they try to resist the behavior. When assessing behavior in clinical practice, we suggest that in the case of repetitive behavior, a general feeling that one *has to* perform the behavior, together with overall recognition that the behavior is not in line with one’s goal, is sufficient to qualify as compulsive even if the strength of these experiences fluctuates over time.

In research applications the three criteria: (1) feels as if one *has to* perform the behavior (2) is repetitive (3) one is aware that it is not in line with one’s goal, can be used to operationalize compulsive behavior. We realize that the operationalization of these criteria to an experimental design is not clear-cut. For instance, the incorporation of the phenomenological criterion (1) may require a questionnaire in human research and may not be translatable to animal models. However, there are inevitably limitations in the extent to which experimental operationalization (and especially animal models) captures clinical phenomena. In this aspect compulsive behavior is not different from other psychiatric phenomena such as depression or hallucinations. A clear definition can help to explicitly point out these differences in order to interpret the findings and translate it back to real world implications. For instance, one operationalization of compulsive behavior in animal models involves ongoing habitual (non-goal directed) behavior despite negative consequences (such as a foot shock) (Hopf & Lesscher, 2014). This operationalization could be viewed as a translational interpretation of the criteria (2) repetitive behavior, and (3) that is not in line with one’s overall goal. Since this model does not test whether the behavior is accompanied by a feeling that one has to perform it, it is possible that the resulting animal behavior is accompanied by different motivations or feelings than compulsive behavior of patients. The awareness of this limitation will prevent an overinterpretation of the results while the model is still useful to test certain aspects of compulsive behavior.

Having a single definition for compulsive behavior will make its meaning more precise and explicit, and therefore more transferable and testable across clinical and non-clinical populations. The purpose of a definition is to specify what to include and exclude under a label. This definition excludes certain types of behaviors like bedtime rituals for young children or stereotyped behavior that does not directly conflict with the person’s goals. Although certain aspects may be shared with the compulsive behaviors under our definition, the inclusion of these behaviors would dilute the concept of compulsive behaviors while other more appropriate labels are available (i.e., ‘stereotypy’ and ‘ritualistic’). However, the current definition still includes a wide variety of behaviors in psychiatry and psychology, behaviours as different as counting calories on food packages, repeatedly washing hands and drinking alcohol. Whether these behaviors share fundamental mechanisms that validate studying compulsive behavior as a cross-diagnostic dimension requires further investigation.
